# Risk factors for mortality after hip fracture surgery in Japan using the National Database of Health Insurance Claims and Specific Health Checkups of Japan

**DOI:** 10.1007/s11657-023-01293-z

**Published:** 2023-07-07

**Authors:** Yuki Nishimura, Yusuke Inagaki, Tatsuya Noda, Yuichi Nishioka, Tomoya Myojin, Munehiro Ogawa, Akira Kido, Tomoaki Imamura, Yasuhito Tanaka

**Affiliations:** 1 Department of Orthopaedic Surgery, Nara Medical University, Kashihara, Japan; 2 Department of Rehabilitation Medicine, Nara Medical University, Kashihara, Japan; 3 Department of Public Health, Health Management and Policy, Nara Medical University, Kashihara, Japan; 4 Department of Sports Medicine, Nara Medical University, Kashihara, Japan

**Keywords:** Hip fracture, National database, Health insurance claim database, Mortality, Risk factors

## Abstract

***Summary*:**

We investigated the risk factors for mortality of hip fracture in the elderly using the National Database of Health Insurance Claims in Japan, and survival was significantly related to sex, age, fracture type, surgical procedure, delayed operative date, comorbidities, blood transfusions, and pulmonary embolism.

**Purpose:**

Hip fracture is the most common fracture in the elderly and is known to have a high mortality rate. In Japan, to the best of our knowledge, no studies have reported on mortality risk factors for hip fracture using nationwide registry databases. This study aimed to determine the number of occurrences of hip fracture and factors that increase mortality using the National Database of Health Insurance Claims and Specific Health Checkups of Japan.

**Methods:**

This study included extracted data from patients who were hospitalized and underwent surgical treatment for hip fracture between 2013 and 2021, using a nationwide health insurance claims database in Japan. Patient characteristics, such as sex, age, fracture type, surgical procedure, delayed operative date, comorbidities, blood transfusions, and pulmonary embolism, were tabulated to obtain 1-year and in-hospital mortality rates.

**Results:**

Both 1-year and in-patient survival were significantly lower in men, older patients, patients who underwent surgery after 3 days of admission, and patients with trochanteric and subtrochanteric fractures, internal fixation, more preoperative comorbidities, blood transfusions, and pulmonary embolism.

**Conclusions:**

Survival was significantly related to sex, age, fracture type, surgical procedure, delayed operative date, comorbidities, blood transfusions, and pulmonary embolism. As the number of male patients with hip fracture will increase with the aging of society, medical staff must provide sufficient information before surgery to avoid postoperative mortality.

**Supplementary Information:**

The online version contains supplementary material available at 10.1007/s11657-023-01293-z.

## Introduction

Hip fracture is one of the most common fractures in orthopedic surgery. Most patients with a hip fracture undergo surgery, such as internal fixation, hemiarthroplasty, or total hip replacement. As the world’s population grows and ages, the number of hip fractures is expected to increase [1]. Globally, 1.7 million individuals had hip fractures in 1990, and this number is expected to increase to 6.3 million by 2050 [2]. Furthermore, hip fracture is associated with high mortality and morbidity in the elderly population [3]. The age- and sex-adjusted 1-year mortality rate for hip fracture is estimated to be approximately 20–30%, and the 30-day mortality rate for hip fracture is estimated to be 5–10% [4]. Various studies have reported on the risk factors for hip fracture, including aging, male sex, and complications [5–7]. There are several studies that have used the national registry database or a claim database to determine mortality risk factors for hip fracture [8, 9]; nevertheless, there are no observational studies with fewer omissions based on national all-source data of 100 million people. And in Japan, local reports are predominant, and no such studies using nationwide registry databases have been reported.

In Japan, the National Database of Health Insurance Claims and Specific Health Checkups of Japan (NDB) was established in 2009. Moreover, Japan has a national health insurance system, and the NDB has the advantage of covering most of the population’s data. It is an all-insurance medical survey that stores almost all the data on medical treatment received by 120 million Japanese citizens. Previous studies analyzing the NDB showed diverse sample sizes. This study successfully analyzed a full-size NDB covering almost all Japanese medical examination data, and also incorporated techniques for individual tracking beyond transfers and relocations [10]. Therefore, this study achieved the largest sample size of hip fracture patients with smallest selection bias compared to previous studies. Japan has the highest aging rate worldwide, with 30% of the population expected to be aged 65 or older by 2025 [11]. Because Japan is leading the world in terms of having an aging society, other countries with aging societies would benefit from comprehensive data collection and research.

This is the first study to investigate hip fracture trends and mortality risk factors by analyzing almost all Japanese patients with hip fracture using the NDB, which is one of the largest claims databases in the world.

## Materials and methods

### Data sources

We extracted data from the NDB of the Department of Health of the Ministry of Health, Labour and Welfare, Japan. Patient tracking/aggregation was performed using our patient tracking technology [10]. From the NDB, hospitalized patients who underwent internal fixation, hemiarthroplasty, or total hip replacement for a first hip fracture during the 8-year period from April 2013 to March 2021 were selected. The at-risk period was defined as the period up to the month of the last medical practice. This study was conducted in accordance with the World Medical Association Code of Ethics on Experiments on Human Subjects (Declaration of Helsinki) and was approved by the Ethics Committee of Nara Medical University (No. 2831). As the data were anonymized, the requirement of obtaining informed consent from individual patients was waived. The database comprised information on procedure codes, procedure application dates, admission dates, International Classification of Diseases and Related Health Problems, Tenth Edition (ICD-10) codes, ICD-10 code documentation dates, and age category codes. Hip fracture was selected from ICD-10 codes for the following three conditions: cervical fracture (ICD-10, S7200), trochanteric fracture (ICD-10, S7210), and subtrochanteric fracture (ICD-10, S7220). Patients younger than 65 years and those with open fractures (ICD-10, S7201, S7211, and S7221) were excluded from the extracted data. All analyses were performed using anonymized data by the NDB.

### Covariates

The number of patients operated on for hip fracture from the NDB was categorized by sex (man and woman), age group (5-year age groups starting at age 65–69 and ending at age 95 or older), fracture type (cervical, trochanteric, and subtrochanteric), procedure (internal fixation, hemiarthroplasty, and total hip replacement), date of surgery after admission (day 0, 1, 2, 3 or later), and the Charlson comorbidity index (CCI) [12] at admission. The CCI was identified from ICD-10 codes based on previous literature [13]. Furthermore, patients who had a blood transfusion or a pulmonary embolism were also categorized.

### Statistical analysis

We used death identification logic in our analysis of the NDB, as described previously [14]. The Kaplan–Meier life table method was used to calculate the 1-year survival rate per month after hip fracture between April 2013 and March 2021. Graphs were created to compare crude survival rates at 1 year from admission by sex, age group, fracture type, procedure type, date of surgery, CCI score (0, 1, 2, 3, 4, 5, or ≥6), blood transfusion (yes or no), and pulmonary embolism (yes or no). Log-rank tests were performed to compare the groups, and factors that increase mortality were analyzed by estimating the adjusted hazard ratio (aHR) and its 95% confidence interval using Cox proportional hazards regression. In addition to 1-year mortality, the number of deaths and mortality rates during hospitalization were analyzed. All analyses were performed using IBM SPSS v.28.0 for Windows (IBM Institute, Inc., Cary, NC, USA), and a significance level of 5% was adopted.

## Results

### Patients

**Fig. 1:**
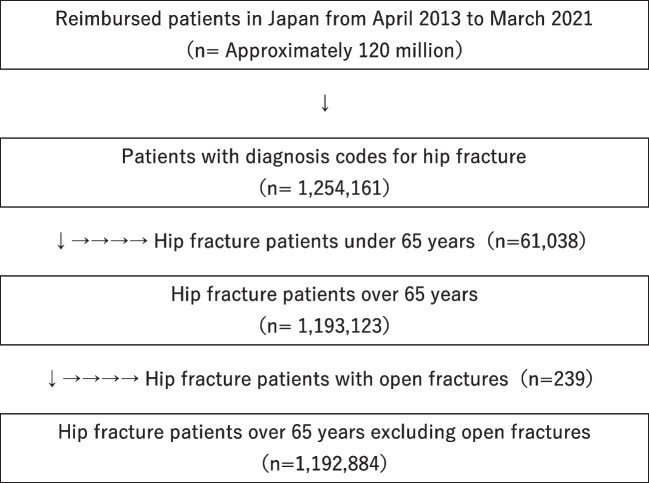
Case selection, identification, and exclusions

The overall number of hip fractures was 1,192,884 (men, 249,747; women, 943,137), excluding 61,038 patients under 65 years and 239 patients with open fractures (Fig. 1). As shown in Table 1, the age range between 85 and 90 years was the most common for both men and women, accounting for 25.2% of men and 28.1% of women. Cervical fractures were the most common type of fracture, slightly more common than trochanteric. Internal fixation was the most common among operation types, accounting for 60% of male patients and 63.5% of female patients. More than half of the patients (men, 62.7%; women, 59.9%) underwent surgery on day 3 or after. A CCI of 2 for men and 1 for women was most common. The percentage of patients with CCI 6 or higher was 15.7% for men compared to 6.9% for women, with a trend toward more comorbidities in men.

**Table 1:** Patient characteristics

	No. (%)		
Characteristic	Total (*N*=1,192,884)	Men (*n*=249,747)	Women (*n*=943,137)
Age, years			
Mean (SD)			
65–69	51,737 (4.3)	17,122 (6.9)	34,615 (3.7)
70–74	83,782 (7.0)	25,500 (10.2)	58,282 (6.2)
75–79	149,808 (12.6)	40,614 (16.3)	109,194 (11.6)
80–84	260,451 (21.8)	59,950 (24.0)	200,501 (21.3)
85–89	328,373 (27.5)	63,005 (25.2)	265,368 (28.1)
90–94	232,733 (19.5)	34,054 (13.6)	198,679 (21.1)
≥95	86,000 (7.2)	9502 (3.8)	76,498 (8.1)
Fracture type			
Cervical	621,640 (51.1)	137,761 (54.2)	483,879 (50.3)
Trochanteric	569,157 (46.8)	111,951 (44.0)	457,206 (47.5)
Subtrochanteric	24,875 (2.0)	4397 (1.7)	20,478 (2.2)
Operation type			
Internal fixation	750,426 (62.9)	150,120 (60.1)	600,306 (63.6)
Hemiarthroplasty	428,921 (35.9)	96,924 (38.8)	331,997 (35.2)
Total hip replacement	14,549 (1.2)	2918 (1.1)	11,631 (1.2)
Operation day			
Day 0	81,545 (6.8)	16,001 (6.4)	65,544 (6.9)
Day 1	203,908 (17.1)	40,464 (16.2)	163,444 (17.3)
Day 2	185,187 (15.5)	36,555 (14.6)	148,632 (15.8)
Day ≥3	722,244 (60.5)	156,727 (62.7)	565,517 (60.0)
Charlson comorbidity index score			
0	191,361 (16.0)	31,249 (12.5)	160,112 (17.0)
1	265,640 (22.3)	41,858 (16.8)	223,782 (23.7)
2	248,177 (20.8)	44,661 (17.9)	203,516 (21.6)
3	185,957 (15.6)	39,617 (15.9)	146,340 (15.5)
4	122,551 (10.3)	31,007 (12.4)	91,544 (9.7)
5	74,231 (6.2)	21,905 (8.8)	52,326 (5.5)
≥6	104,967 (8.8)	39,450 (15.8)	65,517 (6.9)

### Complications

As shown in Table 2, 89,814 men and 395,926 women received blood transfusions during hospitalization, with percentages of 36.0% and 42.0%, respectively. A total of 1268 men and 6167 women were affected by pulmonary embolism, with percentages of 0.5% and 0.7%, respectively.

**Table 2:** Patient complications

	No. (%)		
Complication	Total (*N*=1,192,884)	Men (*n*=249,747)	Women (*n*=943,137)
Transfusion			
Yes	485,740 (40.7)	89,814 (36.0)	395,926 (42.0)
None	707,144 (59.3)	159,933 (64.0)	547,211 (58.0)
Pulmonary embolism			
Yes	7435 (0.6)	1268 (0.5)	6167 (0.7)
None	1,185,449 (99.4)	248,479 (99.5)	936,970 (99.3)

### Mortality and related risk factors

**Fig. 2:**
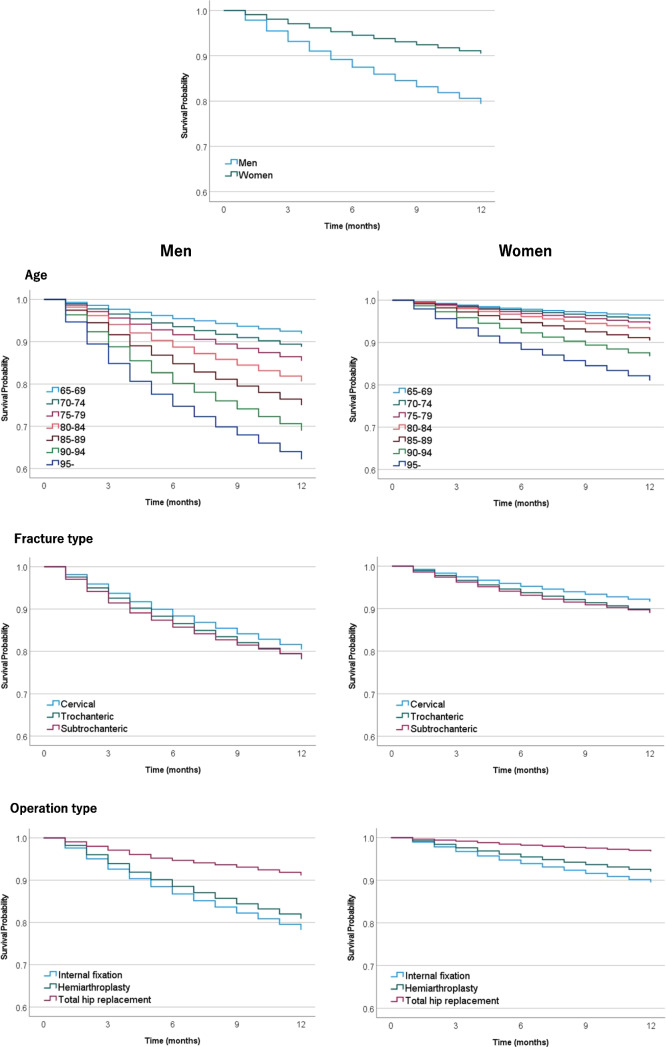

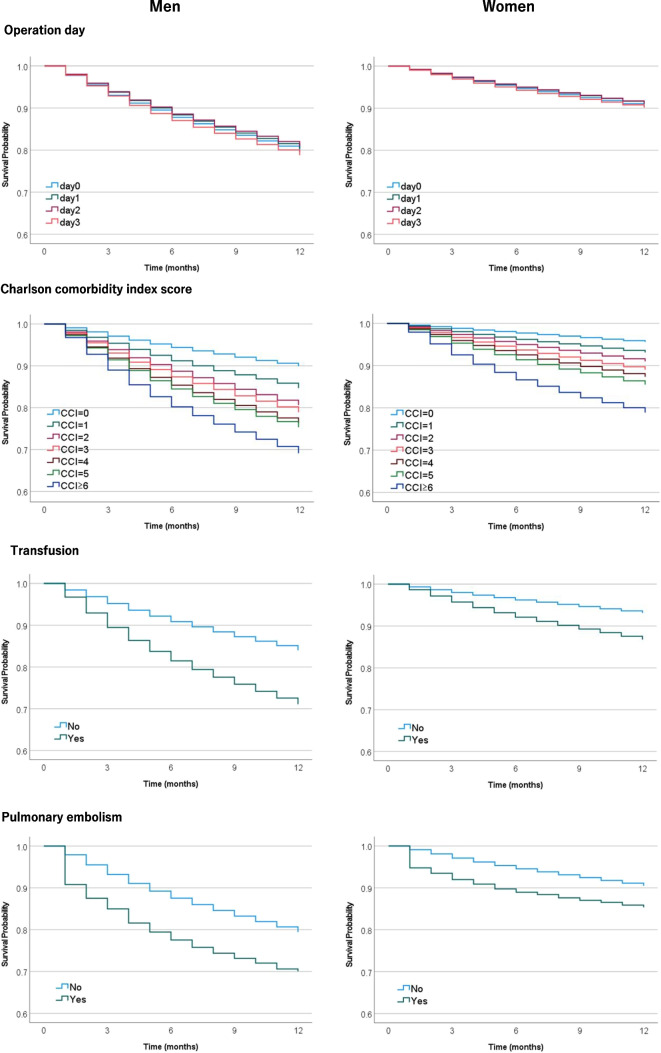
Survival for 1 year after hip fracture in each group

Figure 2 compares 1-year survival rates for hip fractures by sex, age, fracture type, procedure type, date of surgery since admission, CCI, blood transfusion, and pulmonary embolism. Survival rates were significantly lower in men, the oldest group (≥95 years), the trochanteric and subtrochanteric group, the internal fixation group, the late operation group, the highest CCI group (CCI ≥6), the group with blood transfusions, and the group with pulmonary embolism. Table 3 shows the results of the Cox regression analysis. Independent factors that significantly increased the hazard ratio for mortality were as follows: male sex (aHR, 2.29; 95% CI, 2.26–2.32), older age (aHR, 5.93; 95% CI, 5.67–6.19 for age ≥95 vs. age 65–69), fracture type (aHR, 1.24; 95% CI, 1.23–1.26 for trochanteric fracture vs. cervical fracture), internal fixation procedure (aHR, 0.79; 95% CI, 0.78–0.79 for hemiarthroplasty vs. internal fixation), delayed operative date (aHR, 1.03; 95% CI, 1.00–1.05 for day ≥3 vs. day 0), high CCI (aHR, 4.41; 95% CI, 4.31–4.52 for CCI ≥6 vs CCI =0), blood transfusion (aHR, 1.98; 95% CI, 1.96–2.00), and pulmonary embolism (aHR, 1.61; 95% CI, 1.52–1.71). Mortality during hospitalization was also significantly higher in men, the older group, the trochanteric and subtrochanteric group, the internal fixation group, the late operation group, the high CCI group, the group with blood transfusions, and the group with pulmonary embolism. Moreover, the mortality rate was significantly higher, especially in men with age ≥95 years (7.9%), subtrochanteric fractures (5.2%), CCI ≥6 (6.6%), blood transfusions (6.3%), and pulmonary embolism (12.7%) (Table 4).

**Table 3:** Hazard ratios for 1-year mortality after hip fracture based on multivariable Cox regression

Characteristic	Hazard ratio (95%CI)
Male sex	2.29 (2.26–2.32)
Age, years	
65–69	1 [Reference]
70–74	1.33 (1.26–1.39)
75–79	1.68 (1.61–1.76)
80–84	2.21 (2.12–2.31)
85–89	2.99 (2.87–3.12)
90–94	4.12 (3.95–4.29)
≥95	5.93 (5.67–6.19)
Fracture type	
Cervical	1 [Reference]
Trochanteric	1.24 (1.23–1.26)
Subtrochanteric	1.26 (1.21–1.31)
Operation type	
Internal fixation	1 [Reference]
Hemiarthroplasty	0.79 (0.78–0.79)
Total hip replacement	0.32 (0.29–0.35)
Operation day	
Day 0	1 [Reference]
Day 1	0.93 (0.91–0.96)
Day 2	0.92 (0.90–0.94)
Day ≥3	1.03 (1.00–1.05)
Charlson comorbidity index score	
0	1 [Reference]
1	1.57 (1.53–1.61)
2	2.07 (2.02–2.12)
3	2.46 (2.40–2.53)
4	2.85 (2.77–2.92)
5	3.17 (3.08–3.26)
≥6	4.41 (4.31–4.52)
Transfusion	1.98 (1.96–2.00)
Pulmonary embolism	1.61 (1.52–1.71)

**Table 4:** Number of deaths and mortality rates during hospitalization

Characteristic	No. (%)		
	Total	Men	Women
All	20,946 (2.0)	8236 (3.8)	12,710 (1.5)
Age, years			
Mean (SD)			
65–69	429 (0.9)	202 (1.3)	227 (0.7)
70–74	828 (1.1)	406 (1.8)	422 (0.8)
75–79	1839 (1.4)	928 (2.6)	911 (0.9)
80–84	3734 (1.6)	1756 (3.3)	1978 (1.1)
85–89	6043 (2.1)	2579 (4.7)	3464 (1.5)
90–94	5385 (2.7)	1729 (6.0)	3656 (2.1)
≥95	2688 (3.7)	636 (7.9)	2052 (3.1)
Fracture type			
Cervical	10,048 (1.9)	4237 (3.6)	5811 (1.4)
Trochanteric	10,786 (2.2)	3972 (4.1)	6814 (1.7)
Subtrochanteric	625 (2.9)	199 (5.2)	426 (2.4)
Operation type			
Internal fixation	14,279 (2.2)	5331 (4.1)	8948 (1.7)
Hemiarthroplasty	6603 (1.8)	2875 (3.4)	3728 (1.3)
Total hip replacement	86 (0.7)	40 (1.7)	46 (0.5)
Operation day			
Day 0	1128 (1.6)	417 (3.1)	711 (1.3)
Day 1	2802 (1.6)	1059 (3.1)	1743 (1.3)
Day 2	2618 (1.6)	1024 (3.3)	1594 (1.2)
Day ≥3	14,398 (2.2)	5736 (4.2)	8662 (1.7)
Charlson comorbidity index score			
0	1241 (0.7)	418 (1.5)	823 (0.6)
1	2716 (1.2)	849 (2.3)	1867 (1.0)
2	3675 (1.7)	1293 (3.3)	2382 (1.3)
3	3537 (2.2)	1286 (3.8)	2251 (1.8)
4	2983 (2.8)	1215 (4.5)	1768 (2.2)
5	2096 (3.2)	930 (4.9)	1166 (2.5)
≥6	4698 (5.1)	2245 (6.6)	2453 (4.3)
Transfusion			
Yes	13,069 (3.1)	4861 (6.3)	8208 (2.4)
None	7877 (1.3)	3375 (2.4)	4502 (0.9)
Pulmonary embolism			
Yes	494 (7.7)	135 (12.7)	359 (6.7)
None	20,452 (2.0)	8101 (3.8)	12,351 (1.5)

## Discussion

This study investigated risk factors for mortality after hip fracture surgery. Survival rates were significantly lower in men, the oldest group (≥95 years), the trochanteric and subtrochanteric group, the internal fixation group, the late operation group, the highest CCI group (CCI ≥6), the group with blood transfusions, and the group with pulmonary embolism.

Our study included patients hospitalized and operated on for hip fractures included in the NDB data from 2013 to 2021. Data on 1,192,884 patients were extracted, which is one of the largest number of cases compared to previous studies. Ogawa et al. used the Japanese Diagnosis Procedure Combination inpatient database to report seasonal mortality of hip fracture in 425,856 patients [15]. In this study, the 1-year mortality rate for hip fracture was 20.6% in men and 9.5% in women. This compares to previous reports of 21% and 23.9% for hip fracture patients with similar 1-year mortality rates in men [16, 17]. The lower 1-year mortality rate for women in this study compared to those observed in previous studies may have been due to the longer life expectancy in Japan, as it has more advanced medical technology. The 1-year and in-hospital mortality rates tended to increase with age, with 37.8% of men and 19% of women over 95 years of age dying, and the hazard ratio was 5.93, which was higher than that in the 65–69 age group. Studies on hip fracture mortality risk have also revealed that age is significantly related to mortality [7]. For fracture type, trochanteric and subtrochanteric fractures had similar mortality rates in both sexes, with cervical fractures having a lower mortality rate. Similarly, compared to cervical fractures, trochanteric and subtrochanteric fractures are associated with older age and higher CCI scores [18]. Fracture patterns range from simple to unstable, and patients with unstable fractures may not be able to load immediately postoperatively, and the longer time between surgery and transfer increases complications and mortality [19]. These factors may have contributed to the lower mortality rate in patients with cervical fractures. In addition, because trochanteric and subtrochanteric fractures are generally treated with internal fixation, the mortality rate of internal fixation was higher than that of hemiarthroplasty or total hip replacement. In a randomized clinical trial comparing hemiarthroplasty and total hip replacement in patients with hip fracture, patients who underwent total hip replacement had a 58% reduction in 12-month mortality risk [20]. The patients were matched for age, sex, and pre-fracture activity, suggesting that the total hip replacement procedure itself may reduce postoperative mortality. In light of this, total hip replacement is commonly performed in Japan not only in patients with hip osteoarthritis, but also in patients with numerous daily life activities and relatively young patients with hip fracture.

In terms of surgical delay, if the surgery is performed after 48 hours of hospitalization, the mortality rate at 3 and 12 months after surgery for hip fracture in elderly patients increases [21]. Conversely, other studies have shown no effect on mortality within 30 days [22]. However, there is still no agreement on the relationship between early hip fracture surgery and postoperative mortality [23]. In our study, the 1-year mortality rate was slightly higher in the group of patients who underwent surgery after the third day of hospitalization. This may be due to the fact that the date of injury did not always coincide with the date of admission, such as in cases of fracture during hospitalization. Although there was little difference in 1-year mortality in this study. Shen et al. reported that surgical delay increases postoperative complications such as pneumonia and myocardial infarction [24]; thus, it is presumably better to perform surgery as early as possible. In this study, approximately 60% of both male and female patients underwent surgery after the third day of hospitalization. In Japan, early surgery after a hip fracture injury is recommended, but this has not yet been achieved.

With regard to CCI, mortality increased progressively with increasing CCI scores in both men and women, with a particularly high 1-year mortality rate of 30.9% and an in-hospital mortality rate of 6.6% in the group of men with CCI scores of 6 or higher. Therefore, a high CCI score was identified as a potential risk factor for 1-year mortality in this patient population. Previous reports have shown that higher CCI increases both short- and long-term mortality [25], and this study describes specific mortality rates for each CCI.

Regarding blood transfusions and pulmonary embolism, both significantly increased 1-year and in-hospital mortality. While some studies reported that transfusions increase 1-year mortality by 2.79-fold [26], other studies reported that the difference was not significant when patients were matched for background [27]. These results suggest that patients who receive blood transfusions are also likely to have poor preoperative conditions, such as anemia.

In this study, the 1-year mortality hazard ratio for transfused patients after multivariate analysis was 1.98, suggesting that transfusion itself may be a risk factor for death. As a result, it is advised to be cautious about intraoperative bleeding and to limit postoperative blood transfusions to only when absolutely necessary.

Although past studies have mainly examined risk factors for pulmonary embolism because of its low incidence [28, 29], this is the first study to examine the 1-year and in-hospital mortality rates of hip fracture in cases of pulmonary embolism. In particular, we should be concerned about the prevention of deep vein thrombosis in the perioperative period since pulmonary embolism increases the mortality rate during hospitalization by about four times in both men and women.

Our study found that male patients from various backgrounds had higher 1-year and in-hospital mortality rates. Although osteoporosis usually affects women, the results suggest that male patients with osteoporosis may be more fragile. To our knowledge, no previous study has evaluated risk factors for long-term mortality from hip fracture in the entire Japanese population. Furthermore, this study has very little missing data, so our sample is considered representative of the population in this region.

There are several limitations to this study. First, it is a retrospective study. Second, it was based on an insurance database and does not include patients who were not treated by insurance, such as those who had medical assistance for welfare, compulsory automobile liability insurance, or publicly funded medical care. Finally, several other factors that cannot be extracted from the database may also be associated with higher mortality rates, such as secondary osteoporosis caused by steroids, other drugs, or endocrine disorders. The reason for this is that this database consists of Japanese claim data, and items such as blood test results cannot be determined. Despite these limitations, it is assumed that few people are excluded from the data because most Japanese citizens have medical insurance, and most patients with hip fractures receive treatment through medical insurance. Therefore, further studies are needed to investigate other mortality risk factors.

In conclusion, male sex, older age, fracture type (trochanteric and subtrochanteric fracture), internal fixation procedure, delayed operative date, the number of comorbidities, blood transfusion, and pulmonary embolism were significantly associated with risk factors for 1-year mortality in hip fracture. In addition, men had higher mortality rates than women in all categories. In the future, the number of male patients with hip fractures will increase with the aging of society, and medical staff must provide sufficient information before surgery to avoid postoperative mortality.

### Supplementary information

ESM 1 (XLSX 116 kb)

ESM 2 (PDF 342 kb)

## Abbreviations

NDB: National Database of Health Insurance Claims and Specific Health Checkups of Japan
CCI: Charlson comorbidity index
